# NUT midline carcinomas and their differentials by a single molecular profiling method: a new promising diagnostic strategy illustrated by a case report

**DOI:** 10.1007/s00428-020-02869-7

**Published:** 2020-06-25

**Authors:** Simon Haefliger, Alexandar Tzankov, Stephan Frank, Michel Bihl, Alfonso Vallejo, Juerg Stebler, Juergen Hench

**Affiliations:** 1 Institute of Pathology, University Hospital Basel, Schönbeinstrasse 40, 4031 Basel, Switzerland; 2 Department of Medicine, Cantonal Hospital Baden, Baden, Switzerland

**Keywords:** NUT midline carcinoma, DNA methylation profile, Copy number variation profile, Machine learning

## Abstract

NUT midline carcinoma is an aggressive neoplasm defined by chromosomal rearrangements of the nuclear protein in testis (NUT) gene (*NUTM1*). In this article, we present a strategy to detect this rare tumor through a standard DNA methylation array analysis even when occurring in unusual anatomic sites. We illustrate our approach through a case study in which we detected metastatic spread of a NUT midline carcinoma within a bone marrow biopsy that exhibited histological features of a blastoid, undifferentiated neoplasm. Our strategy builds on molecular data derived from The Cancer Genome Atlas and Gene Expression Omnibus as well as computational strategies adopted from the Brain Tumor Methylation Classifier. It is a combined approach that detects the unusual cell lineage of NUT midline carcinomas and makes diagnostic use of the entity-specific copy number alterations.

**Electronic supplementary material:**

The online version of this article (10.1007/s00428-020-02869-7) contains supplementary material, which is available to authorized users.

## Introduction

NUT midline carcinoma (NMC) is an aggressive neoplasm defined by chromosomal rearrangements of the nuclear protein in testis (NUT) gene (*NUTM1*), which most commonly is fused with genes of the bromodomain and extra-terminal domain family, like bromodomain-containing protein 4 (*BRD4*) and bromodomain-containing protein 3 (*BRD3*) [1, 2]. NMC is characterized by a very poor prognosis with a median survival time of 6.7 months and can occur anywhere along the trunk, most commonly along the midline, with typical sites being the head, neck, and mediastinum [2, 3]. Classical methods to diagnose NMC rely on morphology, immunohistochemistry, and in situ hybridization. Here, we present a novel strategy to detect this rare tumor directly through a standard DNA methylation array analysis. We illustrate our approach through a case study of a NUT midline carcinoma within a bone marrow biopsy, exhibiting histological features of a blastoid, undifferentiated neoplasm.

## Materials and methods

### Illustrative case

**Fig. 1:**
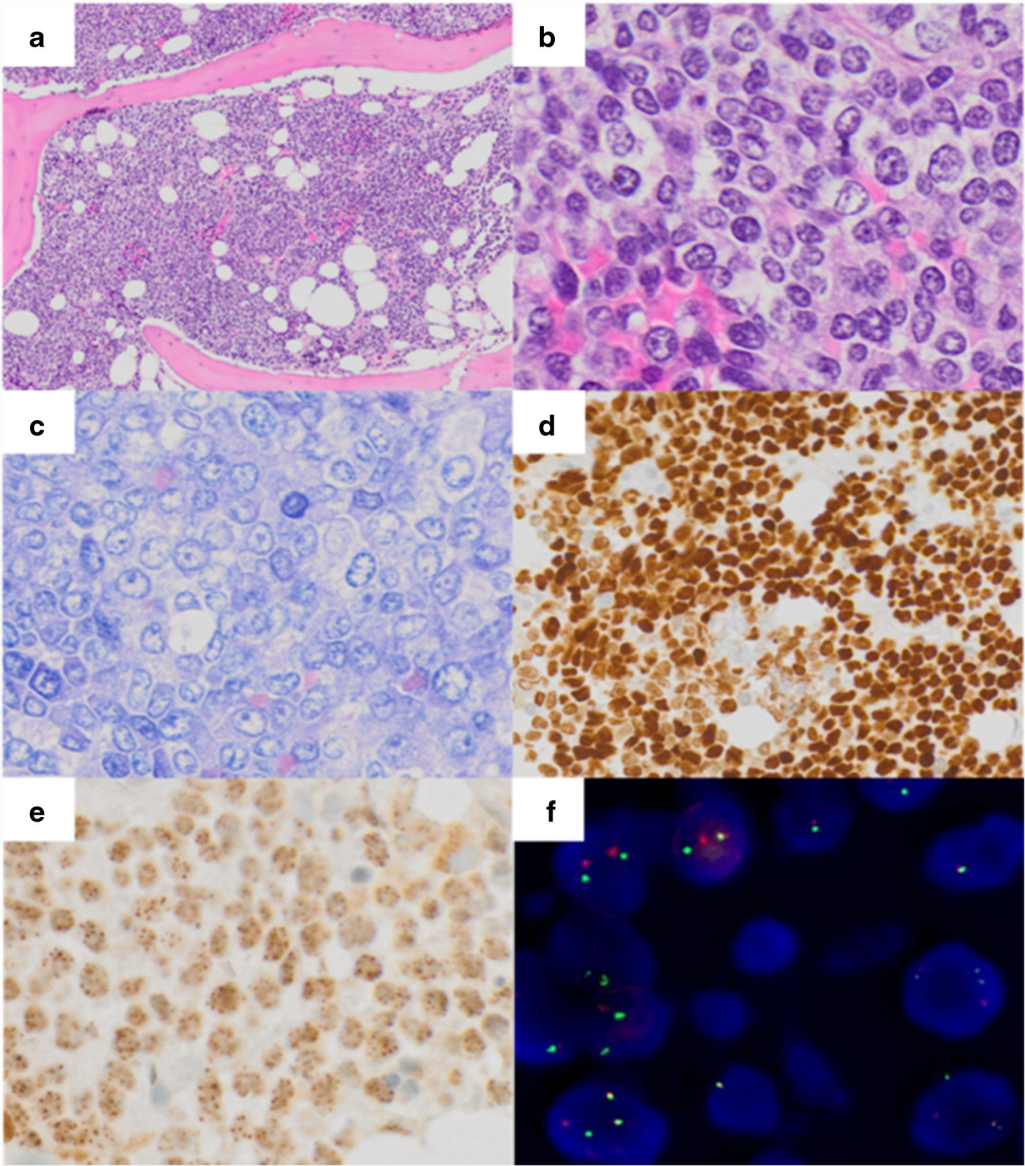
A–E Sheet-like proliferation of monomorphic cells infiltrating the bone marrow (**a**, × 10, Hematoxylin and eosin (HE)). Neoplastic cells feature a blastoid morphology with moderate amounts of clear cytoplasm and round to oval nuclei with vesicular chromatin (**b**, × 40, HE and **c**, × 40, Giemsa), staining for p63 (**d**, × 20, immunoperoxidase) and NUT (**e**, × 40, immunoperoxidase). **f** Dual-color FISH reveals a split-apart of the translocated *NUTM1* gene

A 71-year-old male presented with B-symptoms (night sweats for the last 6 months and weight loss of 10 kg within the last 3 months) and lower back pain lasting for more than 6 months. Magnetic resonance imaging (MRI) revealed multiple lesions in the lungs, mediastinum, kidney, liver, and bones. An externally performed biopsy of the pulmonary mass failed to render a conclusive diagnosis. Subsequently, suspecting a hematolymphoid neoplasm because of accompanying cytopenia, a BM biopsy was performed and showed a diffuse infiltration by cohesive, blastoid appearing medium- to large-sized tumor cells with high mitotic activity (Fig. 1a–c). No squamous or glandular differentiation was found. Suspected differential diagnoses included, among others, melanoma, carcinoma, or (pleomorphic) rhabdomyosarcoma. On immunohistochemistry, the neoplastic cells were positive for CD99 and p63 (Fig. 1d), while remaining negative for ALK, CD3, CD5, CD11c, CD20, CD34, CD79a, CD57, CD117, chromogranin, CK19, CK22, desmin, ERG, Melan-A, HMB45, mast cell tryptase, MPO, MyoD1, PSA, PSAP, S100, TLE1, and WT1 and showed a retained expression of INI-1. Ewing’s sarcoma, mesenchymal chondrosarcoma, and NMC were then considered as differentials. Immunohistochemical NUT protein staining showed granular positivity for NUT in more than 50% of the tumor cells **(**Fig. 1e). Subsequent fluorescence in situ hybridization (FISH) detected a classical *NUTM1* gene break by (Fig. 1f). The patient died 2 months after receiving the diagnosis of NMC.

### DNA methylation array-based computational diagnostic approach

Upon request of the treating oncologists, a DNA methylation array was performed. Approximately 1 μg of DNA, determined by absorption (Nanodrop method) and representing a tumor area of approximately 0.5 mm^2^, was used for the analysis. The tumor cell content was 90%. The DNA methylation profile was then obtained on an Infinium Human Methylation EPIC BeadChip array (Illumina, USA). The resulting IDAT files were processed using the in-house diagnostic toolchain EpiDiP (publicly available at http://www.epidip.org) that allows comparison of a given dataset against currently more than 15,500 reference datasets mostly derived from The Cancer Genome Atlas (TCGA) and Gene Expression Omnibus (GEO). In addition, raw data of diagnostically worked up cases from within our institution and collaborators have been injected into this data lake. The analysis considers both the DNA methylation profile and the copy number variations. This software is primarily written in R (version 3.6.2 as of the time of writing this manuscript) and solely relies on open-source packages. IDAT data, the format provided by Illumina Methylation Array scanners, are parsed through minfi (available from Bioconductor) and normalized using the SWAN algorithm (Bioconductor). Since most reference datasets are from the 450 K methylation array era, we convert all datasets, both from 850 K/EPIC arrays as well as 450 K arrays to an approximately 400 K probe set present in both arrays. Sex chromosomes and cross-reactive probes [4] are excluded. Filtering for the top differentially methylated probes is performed through a standard deviation of all probes across all datasets. Only the top 25,000 probes of this ranking, adopted from the data preparation method used to build the brain tumor methylation classifier, are considered for dimension reduction by uniform manifold approximation and projection (UMAP, R implementation, available from CRAN) [5]. Specimens sharing epigenomic and hence lineage similarities cluster together as demonstrated previously with t-distributed stochastic neighbor embedding (t-SNE) [6].

Genome-wide copy number alterations are read out through conumee (available on Bioconductor) [7]. Copy number plots are calculated for all cases in the reference data lake and made accessible for each case through EpiDiP.

The pan-data-lake UMAP plot is overlaid with tissue type annotations curated in an in-house database and linked to copy number plots for each sample. These data are presented to the pathologist through a Shiny-based web application that we made available for free online [8] (http://www.epidip.org).

**Fig. 2:**
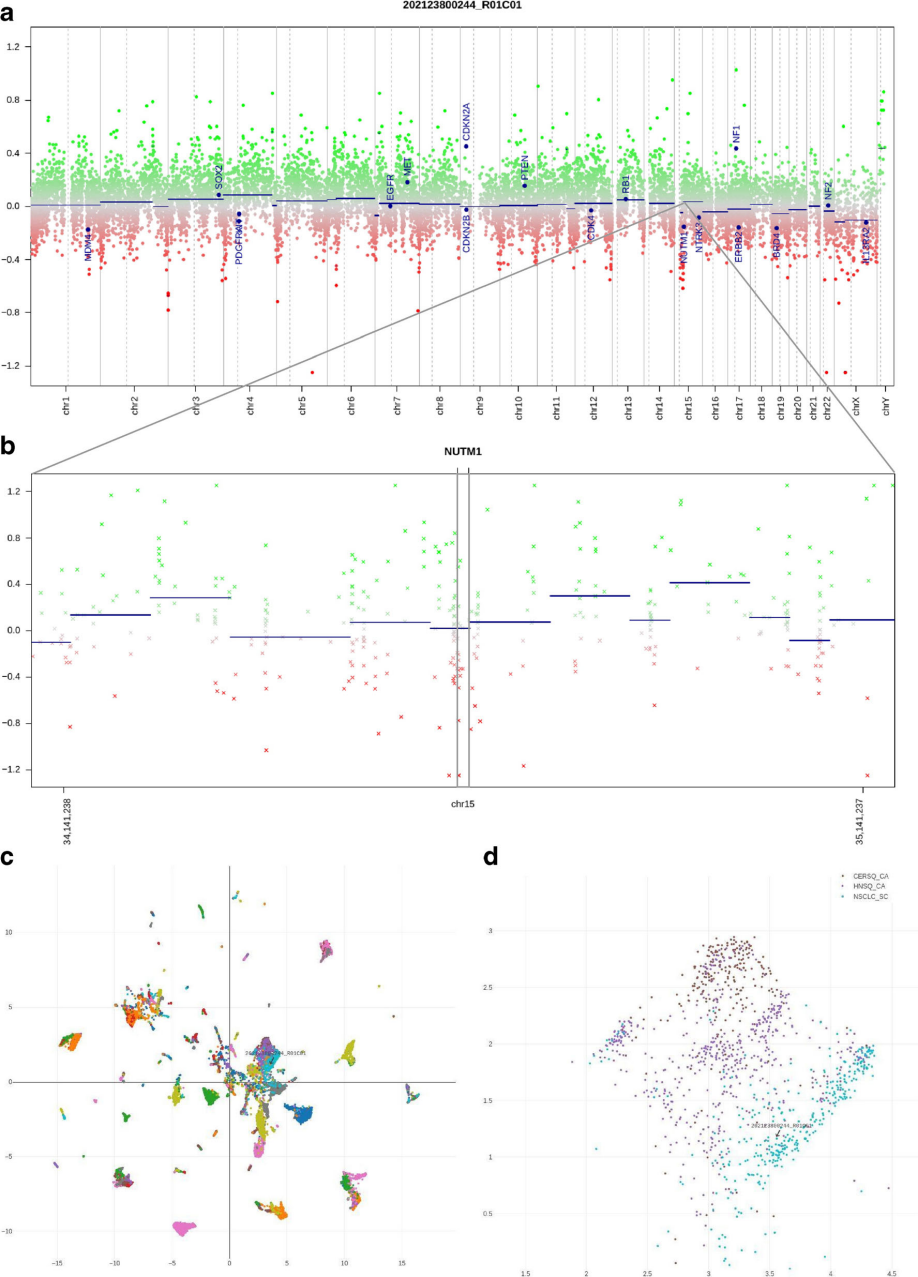
**a** Genome-wide copy number plot calculated from microarray data. The level of the blue dot labeled *NUTM1* represents the average copy number for all *NUTM1* probes. **b** Magnified plot around and including *NUTM1* (center, black box). Note the partial loss (LOH) to the left of the *NUTM1* locus, also including some of the *NUTM1* probes. **c** Overview of the UMAP plot for > 15,000 reference samples; patient sample highlighted by an arrow and array ID. This respective cluster of samples mainly comprises carcinomas of various lineages. **d** Magnification from **c**. The tumor localizes in a sub-cluster containing cancers with squamous cell differentiation. Within this cluster, the similarity is highest to squamous cell cancers (SCCs) of the lung. Abbreviations of the color legend for methylation classes: CERSQ_CA cervical SCC, HNSQ_CA head and neck SCC, NSCLC_SC lung SCC

Upon highlighting a particular diagnostic case through searching for its identifier, neighboring cases can be interrogated for their original annotation and their copy number plots can visually be compared. A user-defined set of genomic loci, typically proto-oncogenes and tumor suppressor genes, is annotated through conumee for improved legibility (Fig. 2a).

## Results

**Fig. 3:**
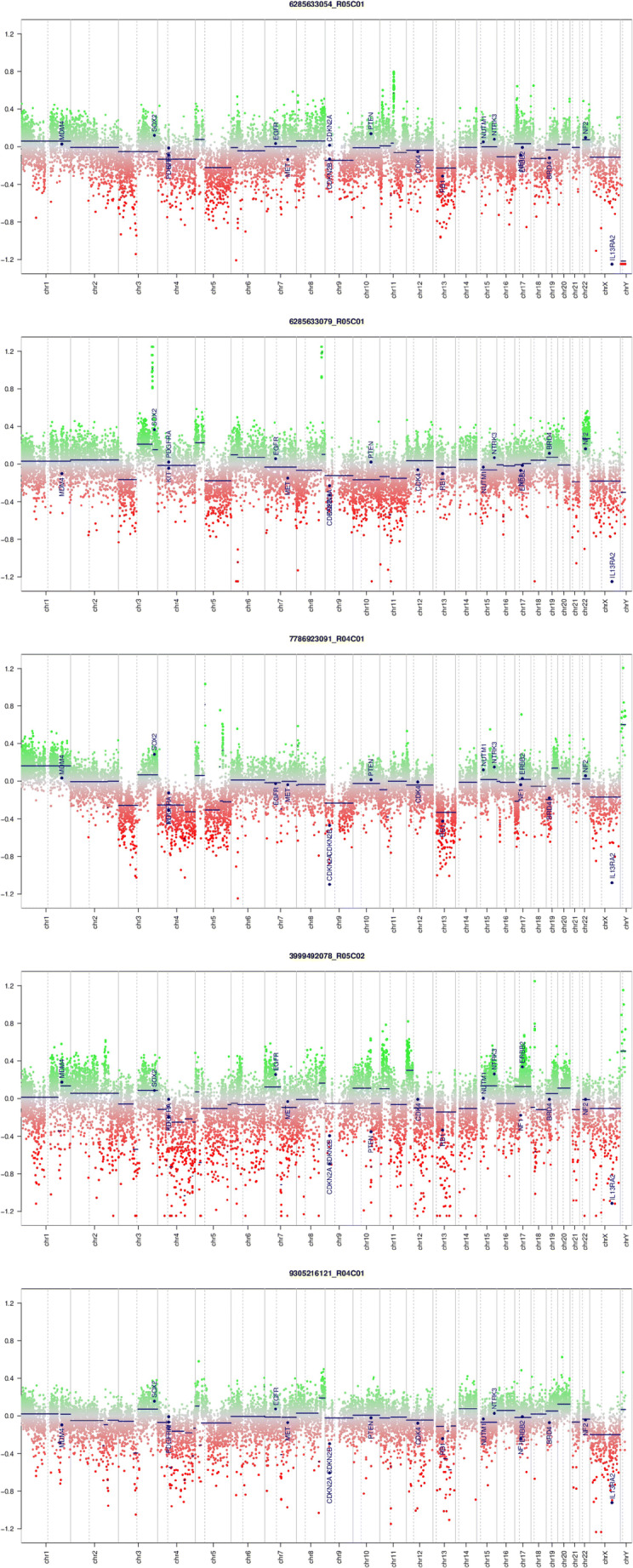
Genome-wide copy number plots calculated from microarray data for the five closest UMAP neighbor cases, all representing squamous cell cancers of the lung. All neighboring cases are TCGA datasets. Note multiple complex copy number alterations in all cases, distinguishing conventional lung squamous cell cancer from the fusion-driven NUT midline carcinoma despite the epigenomic similarities between these entities

Dimension reduction by UMAP indicated a high similarity of the tumor of our illustrative case to squamous carcinomas of the lung (Fig. 2c, d) when compared to over 15,500 reference methylome datasets comprising tumors of diverse differentiation lineages and cells-of-origin. In addition, the chromosomal copy number profile, extracted by conumee, showed a circumscribed loss of heterozygosity (LOH) on chromosome 15q, adjacent to the *NUTM1* locus. The raw array data are available in Supplementary File 1. When invoking the copy number profiles of the five closest neighboring cases, they show the rather disrupted genomes typical of mutagenesis-induced squamous cell cancers (Fig. 3), making this case rather different from common pulmonary squamous cell carcinoma.

## Discussion

The histology of NMC corresponds to an undifferentiated tumor that may or may not exhibit squamous cell carcinoma features [2]. The presence of so-called abrupt foci of keratinization is considered a hallmark of NMC [9]. Another distinctive feature is the cellular monomorphism of the poorly differentiated component, contrasting with the highly atypical, polymorphic tumor cells seen in other dedifferentiated carcinomas [10]. The appearance of NMC overlaps with other poorly differentiated small blue round cell tumors such as Ewing’s sarcoma or acute leukemia [11, 12]. Classical diagnostic methods rely on morphology, immunohistochemistry, and FISH. Classical squamous cell carcinomas often have complex karyotypes and a high mutation load presumably caused by long-term exposure to mutagens such as ultraviolet light, tobacco, or alcohol [2]. In this regard, NMC is clearly different, as this tumor is likely driven by its typical single-gene rearrangement as suggested by the otherwise minor cytogenetic aberrations. Indeed, our analyses revealed a circumscribed deletion involving parts of chromosome 15q, adjacent to the *NUTM1* locus (Fig. 2a, b), and a *NUTM1* break by FISH (Fig. 1f).

Even without any prior knowledge of NUT midline carcinoma, the epigenomic and copy number data from our illustrative case are in line with the hypothesis that such tumors feature a squamous cell-like differentiation and a “flat genome” typical of fusion-driven neoplasms with distinct numeric changes found in the vicinity of a chromosomal break [7]. We are aware that our strategy we routinely apply to a wide range of neoplasms has so far been tested with only a single NNC case; however, we make available our data and analysis platform for public use with the intention to facilitate the collection of further samples of NUT midline carcinoma. The ultimate aim is to include NMC in future machine learning training datasets making it detectable by same-day DNA methylation/CNV diagnostics based on Oxford Nanopore sequencers, too (ONT, UK) [13]. Meanwhile, we propose our strategy to recognize this tumor type based on methylation array data by manual inspection of methylation data through dimension reduction and concurrent copy number profile visualization. Previously published work such as EPICUP [14] would in such cases report “squamous cell cancer of the lung” which is somewhat correct from an epigenomic point of view but does not provide a helpful hint in the given scenario, rather being misleading. EPICUP never provided the pathologist with accompanying CNV profiles. This diagnostically helpful strategy was originally implemented alongside the Brain Tumor Methylation Classifier [15] but is currently restricted to brain tumors. Hence, we also integrated CNV profiles in our UMAP-based tool.

Methylation analysis readily covers all differential diagnoses brought up in our illustrative case. Our UMAP/CNV tool [8] is, in addition, aware of a broad spectrum of hematolymphoid, epithelial, melanocytic, neuroendocrine, cerebral, and mesenchymal neoplasms all of which are represented through publicly available raw data. In summary, we present an alternative, modern, and timely way to detect NMC by a single microarray-based test.

## Electronic supplementary material

ESM 1 Supplementary File 1 202123800244_R01C01_Red.idat (13.7 MB) - Supplementary File 1a 202123800244_R01C01_Grn.idat (13.7 MB) - Supplementary File 1b Raw EPIC microarray data as obtained from the respective Illumina scanner. Data are stored in two files, a red and a green channel. Information about the file format and how to parse it can e.g. be found in the documentation of the open-source minfi software package on Bioconductor (DOI: https://doi.org/10.18129/B9.bioc.minfi). Source code (all in R) for the custom implementation to generate the UMAP and CNV plots are available from the authors upon request.. (ZIP 13.7 mb)
